# Real-Time Multiobject Tracking Based on Multiway Concurrency

**DOI:** 10.3390/s21030685

**Published:** 2021-01-20

**Authors:** Xuan Gong, Zichun Le, Yukun Wu, Hui Wang

**Affiliations:** 1 College of Computer Science and Technology, Zhejiang University of Technology, Hangzhou 310023, China; 1111712011@zjut.edu.cn (X.G.); 1121712004@zjut.edu.cn (Y.W.); 1111712012@zjut.edu.cn (H.W.); 2 College of Science, Zhejiang University of Technology, Hangzhou 310023, China; 3 School of Artificial Intelligence, Zhejiang Post and Telecommunication College, Shaoxing 312366, China

**Keywords:** single-object tracking, multiobject tracking, tracking-by-detection, real-time, multiway, concurrency

## Abstract

This paper explored a pragmatic approach to research the real-time performance of a multiway concurrent multiobject tracking (MOT) system. At present, most research has focused on the tracking of single-image sequences, but in practical applications, multiway video streams need to be processed in parallel by MOT systems. There have been few studies on the real-time performance of multiway concurrent MOT systems. In this paper, we proposed a new MOT framework to solve multiway concurrency scenario based on a tracking-by-detection (TBD) model. The new framework mainly focuses on concurrency and real-time based on limited computing and storage resources, while considering the algorithm performance. For the former, three aspects were studied: (1) Expanded width and depth of tracking-by-detection model. In terms of width, the MOT system can support the process of multiway video sequence at the same time; in terms of depth, image collectors and bounding box collectors were introduced to support batch processing. (2) Considering the real-time performance and multiway concurrency ability, we proposed one kind of real-time MOT algorithm based on directly driven detection. (3) Optimization of system level—we also utilized the inference optimization features of NVIDIA TensorRT to accelerate the deep neural network (DNN) in the tracking algorithm. To trade off the performance of the algorithm, a negative sample (false detection sample) filter was designed to ensure tracking accuracy. Meanwhile, the factors that affect the system real-time performance and concurrency were studied. The experiment results showed that our method has a good performance in processing multiple concurrent real-time video streams.

## 1. Introduction

Tracking moving targets involves processing and analyzing the video (sequence) images captured by photoelectric sensors and making full use of the information to locate and track the target. Target tracking is also the foundation of computer vision. It has important applications in the field of intelligent monitoring [1], pose estimation [2], motion recognition [3], behavioral analysis [4], automatic driving. For example, in an automatic driving system, target-tracking algorithms need to track cars, pedestrians and animals and predict their positions, speeds, and in the future, perhaps other information. 

Target tracking is usually classified into single-object tracking (SOT) and multiobject tracking (MOT) from the perspective of the number of targets tracked. Usually, MOT or multiple target tracking (MTT) is more complex. The main task of MOT is to locate multiple targets of interest simultaneously in a given video sequence and maintain their identifications and record their trajectories [5]. Simultaneously, MOT needs to solve several key problems: (1) determine the number of targets (usually changing with time) and maintain their respective identification, (2) frequent occlusion, (3) initialization and termination of a track, (4) similar appearance, (5) conflict between multiple objectives [1]. 

In practical applications, such as intelligent video application systems and intelligent safety monitoring systems, target tracking often plays an important role. The result of tracking is often used as the input to a correlation processing module, an intelligent video application system that integrates a target-tracking algorithm. However, a real scene of target tracking is often very complex. The numbers and categories of tracked objects are usually not unitary, for example, multiple object categories include pedestrians, vehicles, and nonmotor vehicles. Usually, tracking algorithms solve some common performance problems, such as occlusion or deformation; the complexity of the algorithms also increases linearly. However, in some practical applications, we must face two basic problems: (1) How to process multiple concurrent real-time video streams effectively with MOT under the condition of limited computing resources and storage resources. For example, one GPU (graphics processing unit) card may be used to process more than twenty video streams with MOT. In this case, if the processing speed of the tracking is too low, the “frame loss” phenomenon will occur frequently, resulting in tracking abnormalities. (2) How to balance real-time tracking and algorithm performance. That is, how to effectively improve real-time MOT, keeping system performance within an acceptable range—this is the emphasis in practice. In actuality, other computer vision algorithms will face the same problem also, such as object recognition [6].

Aims of this paper were as follows: (1) Propose one kind of new MOT framework that supports processing of multiway real-time image sequences. Before that, there was no relevant research. (2) Study how to trade off MOT real-time and algorithm performance and design an MOT algorithm based on detection directly. (3) Open new horizons for researchers in the field of high concurrency and real-time processing for MOT. In addition, we also utilized the inference optimization features of NVIDIA TensorRT to accelerate the deep neural network (DNN) processing in the tracking algorithm. 

## 2. Related Works

The real-time performance of a tracking algorithm is usually very important in practice. For example, if processing speed is 30 fps in an MOT system, then only one video sequence with 25 fps frame rate can be supported, which is not sufficient for satisfying large-scale video access in a practical application. 

In recent years, there has been research on MOT real-time. The research has mainly two aspects: tracking framework research and optimization research based on specific hardware and the toolkit environment.

MOT based on tracking by detection (TBD) has become a topic of research in recent years. Alex Bewley has proposed SORT (simple online and real time tracking) [7] tracking technology; this effectively implements faster RCNN (Region-based Convolution Neural Networks)-based detection and tracking using Kalman filtering [8] and the Hungarian algorithm [9] and improves the tracking speed and accuracy. Its frame rate can reach 260 fps. Nicolai Wojke et al. proposed DeepSort (simple online and real time tracking with a deep association metric) [10] based on SORT; this uses deep features learned in the task of person reidentification [11], a greatly reduced ID switch, and solved the problem of short-term target occlusion. Due to the introduction of deep appearance features for a similarity metric, the speed of tracking is approximately 30% slower than the SORT algorithm. In addition, multiple hypothesis tracking (MHT) [12] facilitates precise target detection by maintaining a small list of assumptions, and it adopts an online training appearance model for each trace hypothesis and branch. These need only some extra operations to efficiently learn to the appearance of the model with the regular least squares method framework. However, the complexity of the algorithm will rise as the hypothesis branch increases, especially in scenes with more tracking targets. The tracking speed is only 0.8 fps. In [13], a CNN-based framework was proposed to apply simple single-target tracking to MOT. In terms of computing efficiency, features of CNN are shared, and individual information of each target is obtained by ROI (Region Of Interest) pooling. Concurrently, a spatiotemporal attention mechanism (STAM) is introduced to control the drift problem caused by the interaction between occlusion and the target. The complexity of the algorithm is relatively high, and the tracking speed is only 0.5 fps with a certain CPU power. In [14], a joint detection and embedding (joint detection and embedding, JDE) mode was proposed that uses a single network to output the detection results and the corresponding appearance embedding together. In contrast, the SDE (separate detection and embedding) [10] method and the two-stage method take resampled pixels (bounding box) and a feature map as features, respectively. The MOTA (Multiple Object Tracking Accuracy) of the method was 64.4% on the MOT16 [15] test sequence, obtaining a running time of 18.8 fps. K. Fang et al. [16] proposed a novel recurrent autoregressive network (RAN), which couples an external memory and an internal memory to capture the history and the characteristics of an object during tracking. The external memory explicitly stores previous inputs of each trajectory in a time window, while the internal memory learns to summarize long-term tracking history and associate detections by processing the external memory. The algorithm has good performance on MOT15 [17] and MOT16 [15] datasets and in highly crowded and occluded scenes. Due to the fact that each object needs a RAN, the system needs more space and time consumption, which is not suitable for high concurrency video processing. In [18], authors solved the problem of motion noise and ambiguities between long ranged objects based on a unified framework. This approach was based on the strategy to join the association in both structural and temporal domains. In terms of processing efficiency, the framework was decomposed into a three-stage scheme within an alternative optimization fashion and the processing time was decoupled from the tracklet length via handling association for tracklets-to-tracks based on motion patterns. The average frame rate can reach 10 fps. In [19], the author combined Siamese structure and random forest (RF) and improved the matching performance and solved existing slow CNN-based tracking issues for MOT via a shared-rule based Siamese structure. The algorithm considers both performance and real-time, the processing speed was 12.4 fps. In [20], a new model TubeTK of end-to-end one-step training was proposed for MOT tasks. It utilizes tubes to encode target’s temporal-spatial position and local moving trail. The algorithm achieves the new state-of-the-art performances compared with other online models. In [21], the author focused on solving the problem of tracking trajectory integrity and introduced an iterative clustering method that generates more tracklets while maintaining high confidence. In terms of motion and appearance, two evaluation networks of motion evaluation network and appearance evaluation network were constructed to learn long-term features of tracklets for association. The whole algorithm mainly focuses on performance improvement, not real-time performance. 

Generally, real-time optimization at the tracking-algorithm level (including the tracking framework) is usually limited, so optimizing acceleration based on a specific hardware and toolkit environment has become a research focus. Some companies, such as NVIDIA and Intel, have developed customized accelerators, such as TensorRT (https://developer.nvidia.com/tensorrt) or a neural-computing SDK. They use images created by TensorFlow or Caffe as input and convert them into an accelerated format of inference hardware [22]. Other scholars have also researched DNN acceleration with a GPU. Paras Jain et al. [23] improved the utilization rate of a GPU in a deep-learning inference load by studying time–space multiplexing technology; their experiments showed that the GPU utilization rate increased by a factor of five with such a strategy. In [24], inference, 16-bit quantization, and a CPU multithread based on TensorRT were used to accelerate image processing and good results were achieved. In the field of embedded navigation, Anish Singhani et al. [25] used NVIDIA TensorRT to accelerate the inferencing of a neural network in mobile robot navigation to improve the navigation performance; TensorRT improved inference performance by a factor of nearly three.

As yet, most research on the real-time performance of MOT is mainly based on one-way video stream or image sequence. These cannot satisfy practical applications. In this paper, we mainly focused on the MOT efficient processing of multiple real-time video streams (or image sequences). The experiment results show that our method has a good performance in processing multiple concurrent real-time video streams.

## 3. Methodology

This section mainly focuses on the study of new MOT framework to support concurrent process of multiway real-time video stream. The new MOT framework was expanded in width and depth based on a tracking-by-detection model. In terms of width, the framework can support the process of multiway video sequences at the same time by introducing batch processing mechanism and multiple tracker instance; in terms of depth, image collectors, dispatchers, and filters of negative samples were introduced to meet system performance requirement. In addition, one kind of real-time MOT algorithm based on directly driven detection was introduced to meet the real-time performance and multiway concurrency ability.

### 3.1. MOT Framework Of Multiway Concurrency

**Figure 1:**
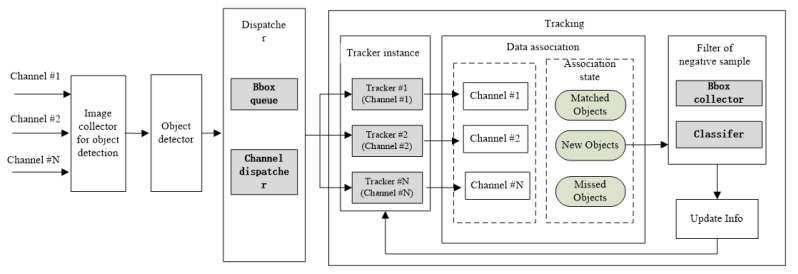
The multiobject tracking (MOT) framework based on multiway concurrency.

In this section, we propose an MOT framework based on a multiway concurrency scenario. The framework mainly consists of four parts: image collector, target detector, task dispatcher, and target tracking, as shown in Figure 1:

In operation, the image collector mainly collects multiway frame sequences and packs each sequence into one image bundle to be used as input to the detector. The bundle consists of a multidimensional vector:

(1) $$B=\left[b_{1},b_{2},\ldots b_{n}\right]\begin{array}{cc} & n\in \max_{channel} \end{array}.$$

Each element of the bundle consists of a four-dimensional vector as shown in Equation (2):

(2) $$b=\left[d_{frame},s_{width},s_{height},c_{index}\right].$$

$d_{frame}$, $s_{width}$, $s_{height}$, and $c_{index}$ denote frame data, frame width, frame height, and channel information, respectively. Multidimensional vector B contains the input parameters for the detector inference. The output state of each detector target is modeled as shown in Equation (3):

(3) $$x=[x_{topleft},y_{topleft},x_{bottomright,}y_{bottomright},l,s].$$

The first four parameters are, respectively, the upper left and lower right corner coordinates of the target bounding box, and the last two parameters are label value and score, respectively. Each target datum (bounding box) will be packaged into a bundle with the appropriate channel identification, that is the channel id: $channel_{i}\in \{0,1,\ldots N\}$, $bundle_{}={\left\{x,channel_{i}\right\}}^{i\in K}$; K is a configurable parameter that determines the size of a bundle. The packaged data will be put into the bounding box queue. 

Considering the unbalanced processing speeds of the detector and tracker, we designed one task dispatcher module. The module is mainly responsible for extracting the target bbox (Bounding Box) from the bbox queue and dispatching the corresponding target bounding box to the tracker according to the channel id. Each image sequence corresponds to one tracker (one tracker instance will be generated for one new image sequence). The operation of the queue is executed in asynchronous mode. 

The tracking part is the focus of this paper. We propose a tracking algorithm based on detection to satisfy real-time performance requirements; one classification filter is designed to filter negative samples, which will improve tracker performance. The tracking part will be introduced in detail in Section 3.3.

### 3.2. Multitarget Detection

At present, in research on multitarget tracking, the framework based on TBD is still dominant. One of the main reasons can be attributed to the application of deep learning in target recognition. The performance of the detector plays an important role in tracking. Mainstream target detection algorithms can be divided into two categories: two-stage, e.g., single shot multiBox detector (SSD) [26], you only look once (Yolo) [27,28] and one-stage, e.g., RCNN [29], faster-CNN [30,31,32,33]. The two categories of detection algorithms have their own advantages and disadvantages. Two-stage detection algorithms divide the detection task into two stages, namely, detection and classification. This kind of algorithm has high accuracy, but its detection speed is slow, so it is not suitable for scenes with strict real-time requirements; one-stage algorithms integrate the two stages into one stage. The real-time performance of this kind of algorithm is better, but its accuracy is slightly worse. Currently, Yolo has been developed to Yolov3 [34], improving the prediction accuracy, especially for small-object recognition on the premise of maintaining the speed advantage. Therefore, Yolov3 was chosen as the detection algorithm in this paper.

Yolov3 adopts an architecture similar to that of feature pyramid networks (FPN) for object detection to realize multiscale prediction (Yolov3 predicts three different scale boxes). In basic image feature extraction, Yolo3 uses 3 × 3 and 1 × 1 convolution to design a network of 53 convolution layers, darknet-53. At the same time, it uses the residual network method to set up shortcut connections between some layers. In addition, due to the application of the multiscale prediction method, the performance of Yolov3 in the detection of small targets is significantly improved. Although the network is larger, the speed is still very fast, and the accuracy is considerable.

**Figure 2:**
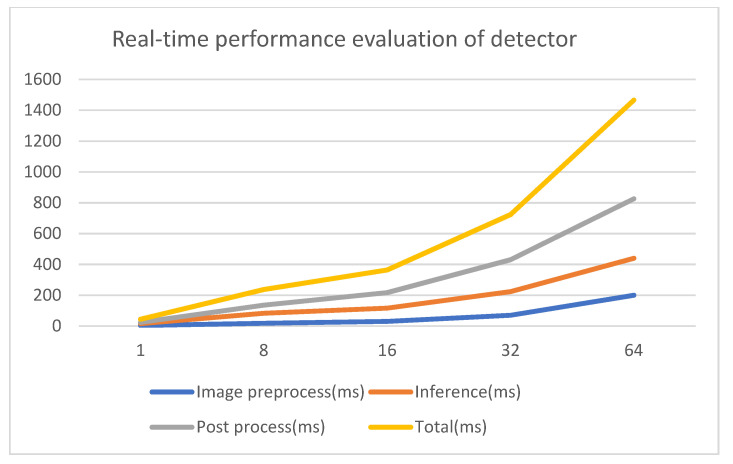
The detector with different batch size (X-axis represents batch size, Y-axis represents time consumption). Blue line indicates image preprocessing, it is related to the operation of the original frame resize; brown line indicates model inferencing for image data; gray line indicates processing of model inference results, including NMSs (nonmaximum suppression); yellow line indicates time consumption of detector.

To study the MOT framework of multiway concurrency, we first obtained the Yolov3 model with the COCO (Common Objects In Context) database based on the TensorFlow deep-learning framework and evaluated the time consumption of the detector with different batch sizes as in Figure 2.

Figure 2 shows that time consumption increases with the increase of batch size. So, it is important to choose the appropriate batch size. Of course, this depends on the overall resources and performance requirement of the system.

### 3.3. MOT Based on Detection Driven

Target-state prediction is a common method for target tracking. Typical target-state prediction algorithms include the Kalman filter and the particle filter [35], which predict the current state and then update it according to the actual state obtained by detection. Typical algorithms include SORT [7] and DeepSort [10]. Data association is an important stage in the process of multitarget tracking. Some reference elaborated the purpose and significance of data association from different point of view [36,37]. In [38], the author showed the role of data association in the usual MOT system in an intuitive graphical way. Data association is closely related to the similarity measurement of targets. Currently, some MOT systems adopt a deep model in the detection and similarity measurement stages [39,40,41,42].

In this paper, we propose one kind of real-time detection-driven MOT algorithm. The algorithm does not adopt a traditional target-prediction method, but associates tracking results and detection results directly (the common method is to associate the prediction results with the detection results) in real-time. To trade off algorithm performance and real-time, one filter of a negative sample based on deep CNN is introduced as well.

#### 3.3.1. Data Association

As mentioned above, the first problem to be solved in multitarget tracking is how to associate the detection results of the current frame and the trajectory set of the tracking target. This is different from single-target tracking, such as MOSSE (Minimum Output Sum Of Squared Error Filter) [43], KCF (Kernel Correlation Filter) [44,45], and DSST (Discriminative Scale Space Tracking) [46,47] (single-target tracking is not related to data association). 

**Figure 3:**
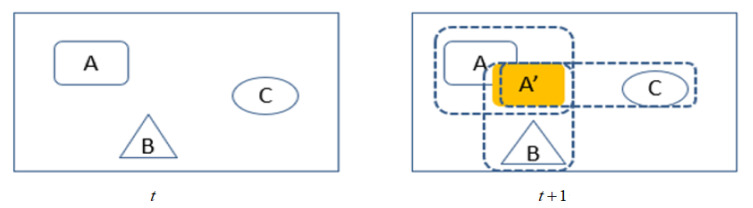
Target matching.

In Figure 3, the tracking targets at time t are A, B, and C. At time t+1, the association to the tracking targets need to establish (here only the association of the target A is drawn). A’ is the position of target A at time t+1 (the detection result at time t+1). A’ needs to match with A at time t, and simultaneously we also need to match other targets to determine if the results of detection and tracking identified the same target. The intersection-over-union (IOU) is usually used to measure the overlap area of two targets. We define a set of tracked targets $T_{m}={\left\{t_{1},t_{2},\ldots t_{m}\right\}}^{m\in N}$, $t_{m}$ to represent each tracked target. The detection target set in the current frame is expressed as $D_{n}={\left\{d_{1},d_{2},\ldots d_{n}\right\}}^{n\in N}$. $d_{n}$ represents the detected target object. We use the Jaccard coefficient to calculate the degree of matching between the detection result $D_{n}$ of the current frame and the tracking target set $T_{m}$. Jaccard coefficient is used to compare the similarity and difference between limited sample sets. Given two sets D and T, Jaccard coefficient is defined as the ratio of the size of the intersection of D and T to the size of the union of D and T (Intersection over Union, IOU). The definition is as follows:

(4) $$J\left(D,T\right)=\frac{\left|D\cap T\right|}{\left|D\cup T\right|}=\frac{\left|D\cap T\right|}{\left|D\right|+\left|T\right|-\left|D\cap T\right|}.$$

These values will be used to build a cost matrix, as shown in Equation (5):

(5) $$C_{m\times n}=\left(\begin{array}{cccc} j_{d_{1},t_{1}} & j_{d_{1},t_{2}} & \ldots & j_{d_{1},t_{n}}\\ j_{d_{2},t_{1}} & j_{d_{2},t_{2}} & \ldots & j_{d_{2},t_{n}}\\ \vdots & \vdots & \ddots & \vdots \\ j_{d_{m},t_{1}} & j_{d_{m},t_{2}} & \ldots & j_{d_{m},t_{n}} \end{array}\right)\begin{array}{cc} & m,n\in \left\{1,2,\ldots ,N\right\} \end{array}.$$

$j_{d_{m},t_{n}}$ represents the Jaccard coefficient between the detected target $d_{m}$ and the tracking target $t_{n}$ in the current frame. The data association is generated by the Hungarian algorithm as shown in Equation (6):

(6) $$L_{\left(d_{index},t_{\;index}\right)}=H(C_{mxn})\;\;\;\;\;\;m,\;n\in \{1,2,\ldots ,N\}.$$

The function H is a linear assignment function that returns the matrix $L_{\left(d_{index},t_{\;index}\right)}$. Each row of the matrix establishes a matching relationship between the detection target and the tracking target. 

**Figure 4:**
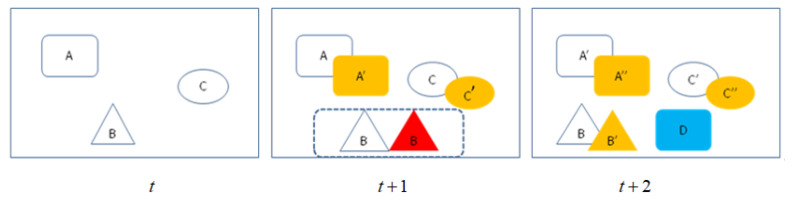
Data correlation scenario in multitarget tracking (the yellow represents target tracked correctly; red represents target of last frame was not detected at current frame; blue represents new target). At time t, tracklet includes three targets; At time t+1, target B wasn’t detected; at time t+2, new target D was detected.

The target-tracking process may be affected by some unknown external factors that may lead to abnormal tracking, such as illumination, occlusion, and data association problems caused by missing detection of the detector. Figure 4 shows missing detection and new target appearing scenarios in multitarget tracking.

As shown in Figure 4, the three objects A, B, and C can be considered as tracking targets at time t. At time t+1, A and C are detected correctly and correspond to A’ and C’ respectively, but object B in the current frame is undetected; this means that the established association relationship failed with tracking object B at time t. We can obtain the target of missing detection $D_{miss}$ via Equation (7):

(7) $$D_{miss}={\left\{d_{object}|d_{object}\in T_{m},d_{object}\notin L_{t_{index}}\right\}}^{m,index\in N}.$$

At time t+2, the three tracking objects, A, B, and C, were detected and associated correctly, but object D was a new target. The new emerging target $D_{new}$ can be obtained by Equation (8):

(8) $$D_{new}={\left\{d_{newobject}|d_{newobject}\in D_{n},d_{newobject}\notin L_{d_{index}}\right\}}^{n,index\in N}.$$

#### 3.3.2. Object Creation and Disappearance

In the process of multitarget tracking, it is necessary to solve the matching problem between the detection result of the target and the trajectory of the target; this can also be regarded as target reidentification. Therefore, how is a tracked target trajectory maintained? The usual TBD method needs to adjust the result of a prediction algorithm by detecting the result, and then update the result to the nearest target trajectory. Meanwhile, some targets will appear, and some will disappear. The life cycle of an object begins upon entering the lens and ends on exiting the lens. During this period, the object may be occluded or missed. However, maintenance of the target trajectory is crucial in the whole tracking process. In this paper, the detection results of the first frame (for example, at time t) were taken as the basic target set, and the detection results of the next frame (for example, at time t+1) were associated with the target track set. A new target was assigned a new target id, and its motion information was initialized. In addition, if the number of missed detection targets exceeded a threshold value $A_{loss}$ (this threshold value can be set based on a priori knowledge, such as 10 or 15), then we decided the target had disappeared, and the target trajectory set was updated at the same time.

#### 3.3.3. Filter of Negative Sample

In the MOT framework of multiway concurrency, we introduced one filter of a negative sample to improve tracking accuracy. Next, we will describe the function and real-time performance in detail.

**Figure 5:**
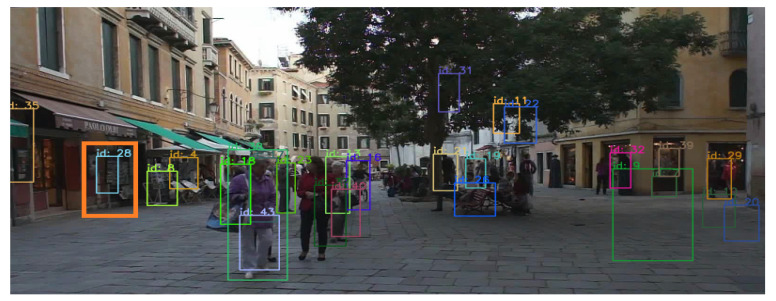
False target detection.

Usually, the quality of the detector has a direct impact on tracking accuracy based on the tracking-by-detection framework, for example, false detection or missed detection. Missed detection does not lead to time consumption of the tracking algorithm, but false detection not only causes time consumption but also affects the accuracy of tracking. However, each target may be related to false detection problems; for example, it is possible that garbage or some road signs, and even a roadside tree are detected as pedestrians as shown in Figure 5 (orange rectangle, raw image was from MOT challenge dataset [15]). Id 28 was detected mistakenly as pedestrians. The false detections cause unnecessary consumption of computing and storage resources and impact tracking performance. 

**Figure 6:**
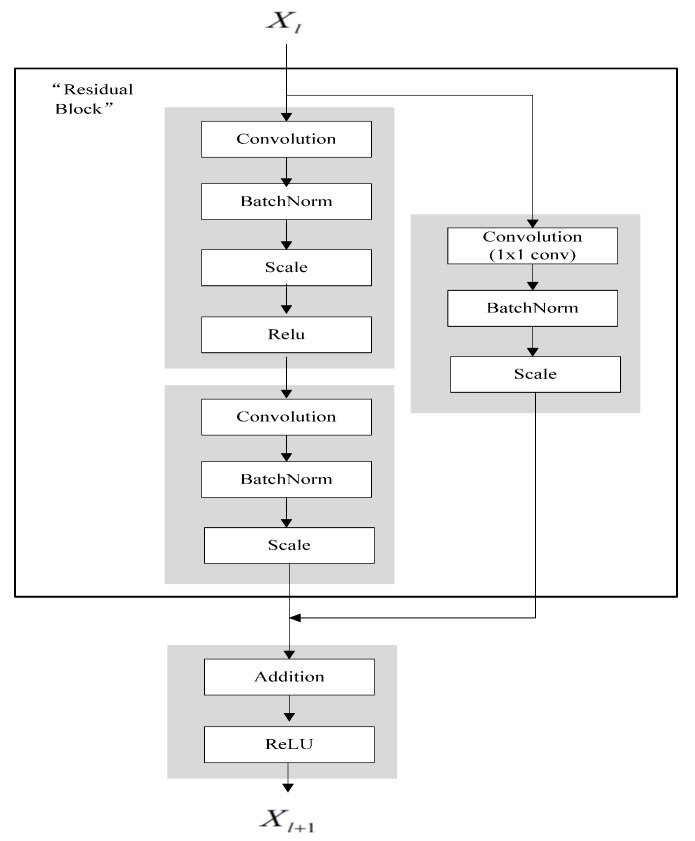
Residual block structure.

To mitigate false detection, we designed a deep CNN filter. In the process of tracking, if there is a new target, the filter is used to check if the target meets the requirement. Currently, the filter supports two categories, vehicles and pedestrians. The structure of the deep network is shown in Table 1:

The network architecture of CNN is composed of a convoluton layer, two pooling layers, four residual blocks, a full-connection layer, and a SoftMax layer. The initial input picture dimension is 196 × 196, and the full connection layer and SoftMax layer are used for category prediction. The residual block structure is as shown in Figure 6.

In a convolutional network, since the number of feature maps of $x_{l}$ and B$x_{l+1}$ is different, 1 × 1 convolution is needed to increase or reduce dimensions. The residual block is expressed as:

(9) $$x_{l+1}=h(x_{l})+F(x_{l},W_{l})$$

where $h(x_{l})=W_{l}^{\prime }x$, $W_{l}^{\prime }$ is the convolutional operation of 1 × 1; $F(x_{l},W_{l})$ is residual portion.

From the perspective of real-time performance, we must consider the filter inference speed and the whole system impact. If multiway concurrency occurred and every frame has many detected results to filter, then system real-time will be impacted; the resource consumption of the system will be linear with the number of targets bounding boxes. We solved this problem from three aspects: (1) we designed a bounding-box collector to pack target bounding boxes of every channel into one batch to be input parameters of the filter; (2) if the filter processes all target bounding boxes, then the computing cost is still high, so we allowed the filter to process only new targets in every frame—missed and matching targets will not be processed by the filter; (3) we fully exploited TensorRt’s inference and optimization characteristics to accelerate the processing speed of the classifier. Based on the three-aspect improvement, resource consumption will be greatly reduced. 

In addition, we found that the bbox queue will overflow after a period of time in multiway concurrency and that the main time consumption is the filter; the experimental average time consumption per frame is approximately 9.93 ms, which cannot meet the real-time requirements of the multiway concurrent MOT system very well.

The above problems are mainly caused by frequent image data copying between the CPU and GPU. The decoded images are stored in the DDR (Double Data Rate) memory of the system and processed by the CPU. When the cropped images (bounding box) are transferred to the filter, the bounding box will be copied to the GPU memory by the CUDA (Compute Unified Device Architecture) during preprocessing. Therefore, we attempted to allocate GPU memory for the image decoded by CUDA, with subsequent image operations implemented in the GPU. Experimentally, the time consumption of the filter processing one frame was approximately 5.97 ms, an obvious improvement.

## 4. Results

In this section, we first introduce the experiment environment, including the hardware and software environments and experiment inputs. Next, in the experiment evaluation part, we mainly compare the resource consumption of the algorithm in the tracking process and compare its performance with similar algorithms. Finally, the experiment results are analyzed and summarized.

### 4.1. Experiment Environment

We used the MOT challenge dataset MOT16 [15] as the data source. The experiment environment included: GTX 2080TI GPU, CUDA version 10.0, cuDNN version 7.4.2 for both toolkits, and the TensorRT version was 10.0.1. Based on the above environment, three groups of experiments were compared to reach experiment conclusions.

According to the MOT system structure, the detection results were packaged into a bounding box bundle as the input to the tracking algorithm (these bundle is first inserted into bbox queue and then dispatched to corresponding tracker by channel dispatcher.). The target bounding box in the bundle could come from different image sequences (that is, a different camera) or the same image sequence; every image sequence was identified by a unique channel id. 

In this experiment, to verify the correctness of the basic function on MOT system, we simulated processing two channels with identical input image sequences, that is, five frames in a bundle were from channel #1, while the other five frames were from channel #2. Bundle size was set as 10. The tracking algorithm processed these two image sequences in parallel (one image sequence was processed by one tracker instance). If the processing is correct, the output of each channel should be the same; that is, the two channels have the same tracking target information.

**Figure 7:**
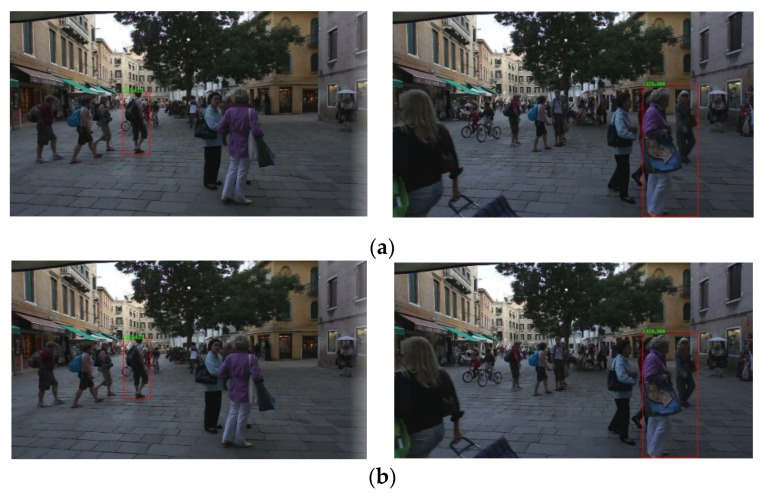
Tracking results of two identical image sequences. MOT16 [15] dataset was used to verify the basic function of tracking system: (**a**) The tracking results of channel #1; (**b**) the tracking results of channel #2.

We only sampled two tracking targets and visualized them as shown in Figure 7. From the output results, it can be observed that the tracking had the same processing results for two identical image sequences, this shows that the logic of the tracking system is correct, which is the basis of subsequent experiments.

### 4.2. Real-Time Evaluation

The detector has a direct impact on the tracking system in terms of real-time and algorithm performance based on the TBD framework, that is the different detector will generate the different impact on MOT system. To objectively evaluate the real-time performance of the tracking part in the paper, we did not consider the detection part.

In the paper, we proposed a MOT framework of multiway concurrency. We also designed a filter of negative sample to improve tracking accuracy. In terms of width of the MOT framework, the number of tracker instance will increase with the increase of concurrency. One tracker instance process one image sequence. One performance issue needed to be considered, that is, does one filter correspond to one tracker instance or multiple tracker instances? Which strategy is the most efficient? According to the different approaches of image data storage and processing in the tracking process, we divided the experiment into the following three groups, in the first two groups’ experiments, we adopted one filter correspond to one tracker instance, but in the third group experiment, we adopted one filter to process the multiple tracker instance in parallel. 

(1) In group one, all of the image data in the bundle were stored in DDR memory (image data were collected by image collector) and the tracker processed the detection results in each frame (one tracker is for only one image sequence) and output the processing result of each frame after processing a bundle.

(2) In group two, we allocated GPU memory for each frame by CUDA memory management function, that is, the image data in the bundle were stored in the pre-allocated GPU memory (image data were collected by image collector) and the tracking algorithm processed the detection results using the same processing flow as the first group experiment.

(3) In group three, the frames in a bundle were allocated the same way as in the second group experiment, but differently from the above two group experiments for bundle processing. In other words, after one bundle was processed, all target information was collected by the bounding box collector, and these targets were processed by the classifier at once.

**Figure 8:**
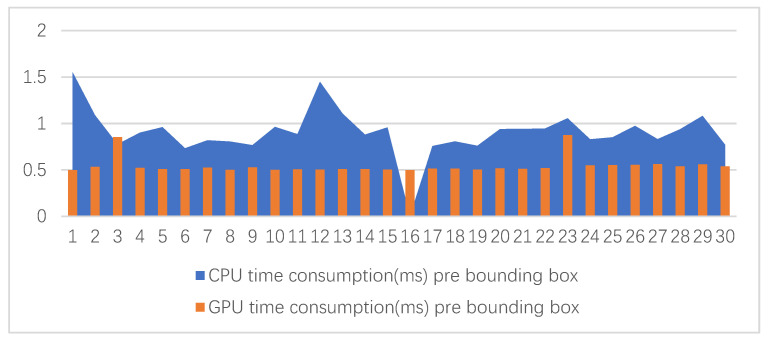
Computational resource consumption of each bounding box.

We have statistics of the classifier time consumption in processing each bounding box for experiments group one and group two (the statistics of only 30 bounding boxes was done), as shown in Figure 8. The x-coordinate is the number of bounding boxes and the y-coordinate is the time consumption of processing a bounding box:

According to Figure 8, the classifier time consumption in the second group experiment was, on average, much less than the first group for processing a bounding box. The classifier is related to GPU preprocess. In the preprocess, image data are copied from external memory to GPU memory in the first group experiment, which consumed more time. The time consumption of three groups of experiments for processing one bundle were 149.05539 ms, 89.57784 ms and 2.65549 ms, respectively. One bundle included the fifteen-frame information from different image sequences. The statistics show the third group experiment improved the second group experiment efficiency by a factor of 33.

**Figure 9:**
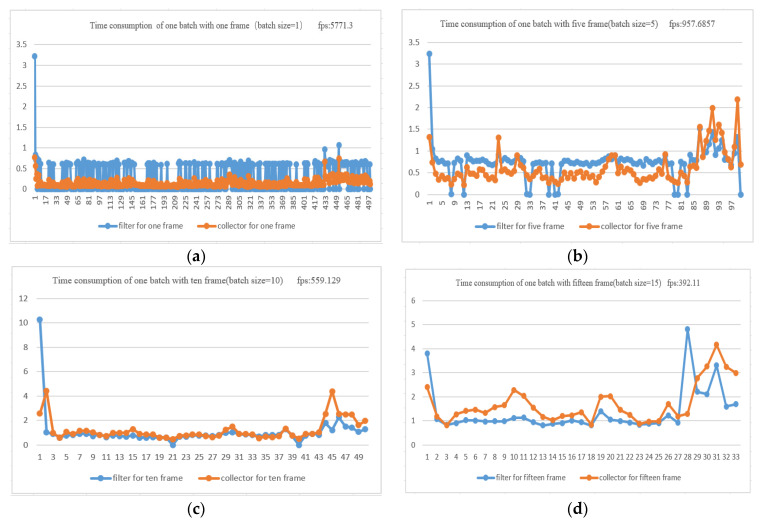
Time consumptions of different batch sizes: (**a**) the batch size was 1; (**b**) the batch size was 5; (**c**) the batch size was 10; (**d**) the batch size was 15.

The classifier is responsible for the image processing in the whole tracking algorithm, which is one of the key factors that impact the system performance. So, we evaluated tracking speed in the third group experiment with different images batch sizes (1,5,10,15) separately (the images of different batch size were used as input to classifier), as shown in Figure 9:

The experimental results show that the tracking speed decreased with the increasing batch size as shown in Table 2, which was caused by the bounding box collector (the collector will crop images to get a bounding box). We will analyze this more deeply in Section 4.3. 

### 4.3. Performance Evaluation

Next, we evaluated the performance of MOT based on MOT challenge dataset MOT16 [15], evaluation indications and definitions as shown in Table 3. 

MOTA is a combination of three errors—false positive, missed targets, and identify switch—it is computed as:

(10) $$\mathrm{MOTA}=1-\frac{\sum_{t}\left(fn_{t}+fp_{t}+idsw_{t}\right)}{\sum_{t}g_{t}}\in \left(-\infty ,1\right]$$

where for frame t, $fn_{t}$ is the number of false negatives (missed targets), $fp_{t}$ is the number of false positives (false detection), $idsw_{t}$ is the number of identity switches, and $g_{t}$ is the number of objects present. The higher MOTA indicates higher tracking accuracy. MOTP (Multiple Object Tracking Precision) measures the error of the predicted bounding boxes and the ground truth. The original formula was:

(11) $$\mathrm{MOTP}=\frac{\sum_{i,t}d_{t}^{i}}{\sum_{t}c_{t}}$$

where for frame t, $d_{t}^{i}$ is the distance between the ground truth and prediction of object i, and $c_{t}$ the number of matches found. 

To be fair, we compared only the key performance indicators; the real times of the algorithms were not compared, because the run environments of the algorithm were different, as shown in Table 4.

**Figure 10:**
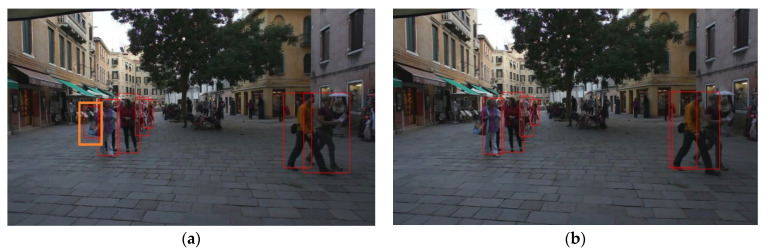
Tracing effect after using filter. (**a**) Before using the filter of negative sample; (**b**) after using the filter of negative sample.

From the above evaluation results, under the premise of high concurrency and real-time, the performance of our method was still acceptable. This is mainly because the filter suppresses the negative samples and ensures the performance of the tracking algorithm. The results after applying the filter are shown in Figure 10 (raw image was from MOT challenge dataset MOT16). 

In the left picture, the detector mistakenly thought the satchel was a person, and in the right picture, the misidentified target was filtered to improve the tracking performance. In our approach, the ID switch was still a bit high, caused mainly by the overlap of some targets or long-term occlusion in the test set, which has not been solved well in this paper.

### 4.4. Discussion

According to the above experiments, the time consumption of tracking depends mainly on two factors: (1) Processing of image data between the CPU and GPU. In the GPU preprocess, every bounding box needs to be copied from external memory to GPU memory for the first group experiment, causing time consumption. (2) In the third group of experiments, the detected objects in a bundle were collected by the collector. This process cropped the image (bounding box) and allocated GPU memory for the image cropped by CUDA, then the filter processed them at once. Table 5 presents statistics of the time consumption of the collector and filter with different batch sizes. According to the statistics, the time consumption of both increased with the increase of batch size.

**Figure 11:**
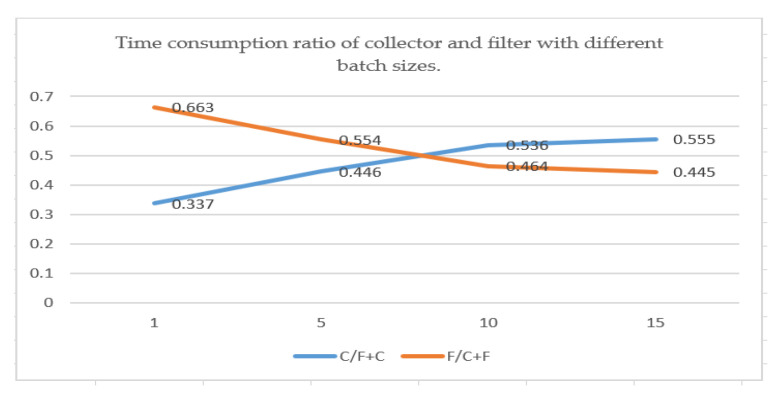
Time consumption ratio of collector and filter with different bundle sizes (X-axis represents batch size, Y-axis represents time consumption ratio).

Further analysis showed that the proportion of time consumption of the collector C/(F+C) increased with the batch size, but the proportion of filter time consumption F/(C+F) decreased gradually with the increasing batch size, as shown in Figure 11. 

In the collector process, the detected bounding box needs to be cropped and stored in GPU memory by CUDA; a larger batch size caused more time consumption. For the filter (classifier), a suitable number of input images causes full filter use of GPU computing resources. From Figure 11, when the number of input images was small, the time consumption ratio of the filter was higher than that of the collector, and just near the cross point, the time consumption ratio of the two was close, so a batch size of 10 can be a suitable value.

Of course, from the memory point of view, the results of the last two groups of experiments caused GPU memory to increase significantly, especially when the number of objects processed increased. In the process, GPU memory is frequently allocated and released, this also causes more time consumption.

In addition, the paper adopted a type of detection-based tracking algorithm; that is, the detection result of the current frame was regarded as a query, the tracking object list was regarded as gallery, and the best match was found through the Hungarian algorithm. The algorithm performed well without long-time occlusion. When there is long-time occlusion, lost-object problems may occur. The long-term occlusion issue is complicated, solving such problems usually means consuming more computing and storage resources, which then impacts the system’s real-time performance. Considering the high concurrency and real time of processing of the tracking algorithm, we traded off the performance of the algorithm and the processing speed. Before that, there was no relevant research. Actually, the balance between the real-time performance of target tracking and algorithm performance in case of high concurrency is a problem worth studying, even if sometimes they are contradictory. How to balance their relationship? There is no definitive answer or metric. In our opinion, when the algorithm meets the real-time requirements of the system, performance loss may be acceptable under the conditions of practice.

## 5. Conclusions

The MOT with a multicamera is a kind of general vision application. In this case, usually there is a critical demand for real-time tracking performance and system concurrency capability. In our paper, we proposed a new MOT framework to support the concurrent process of multiway real-time video stream. The new MOT framework was expanded in width and depth based on a tracking-by-detection model. We then conducted the performance research of MOT based on three aspects of the working model: (1) We proposed one type of simple MOT-based direct detection. In the tracking algorithm, a CNN network was designed as the filter (classifier) to reduce false detection. (2) We designed a batching mechanism to speed up the classifier efficiency in the tracking process. The experimental results showed that the new mechanism generated larger gain. (3) We utilized the inference optimization features of NVIDIA TensorRT to accelerate the filter processing in the tracking algorithm.

The final experiment showed that our implementation and optimization of a real-time multiway concurrent multiobject tracking system can meet the practical scene application requirements. Of course, some issues need to be researched further, for example, the long-duration occlusion caused the target to be lost. Although this issue was solved well by DeepSort, it was slower, so further real-time tracking performance will be impacted. In addition, frequent GPU memory allocation and deallocation will incur more time consumption.

## Figures and Tables

**Table 1:** Network structure of the filter.

The Name	The Kernel Size/Stride	Num Output
1 Conv	7 × 7/2	32
Pool2	3 × 3/2	32
Residual 3	3 × 3/1	32
Residual 4	3 × 3/1, 1 × 1/2	128
Residual 5	3 × 3/1, 1 × 1/2	256
Residual 6	3 × 3/2, 1 × 1/2	128
FC7		2
SoftMax		2

**Table 2:** The tracking speed with different batch size of bounding box.

Batch Size (Concurrency)	fps
1	5771.3
5	957.6857
10	559.129
15	392.11

**Table 3:** MOT evaluation indications and definitions. “↑”: higher scores denote better performance, “↓”: lower scores denote better performance.

Evaluation Indications	Definitions
MOTA↑	The multiple objects tracking accuracy [48]
MOTP↑	The multiple objects tracking precision [48]
MT↑	Ratio of successful tracking target trajectory to real target trajectory
ML↓	Proportion of lost target trajectory to real target trajectory
IDsw↓	The total number of target identity switch during the whole target tracking process
FP↓	The total number of false positives
FN↓	The total number false negatives
Frag↓	The number of times the real trajectory is interrupted

**Table 4:** Multitarget tracking evaluation list. “↑”: higher scores denote better performance, “↓”: lower scores denote better performance.

Methods	MOTA↑	MOTP↑	MT↑	ML↓	IDsw↓	FP↓	FN↓	Frag↓
SORT [7]	59.8%	79.6%	25.4%	22.7%	1423	8698	63,245	1835
DeepSort [10]	61.4%	79.1%	32.8%	18.2%	781	12,852	56,668	2008
MHT_DAM [12]	45.8%	76.3%	16.2%	43.2%	590	6412	91,758	781
POI [49]	66.1%	79.5%	34.0%	20.8%	805	5061	55,914	3093
RAN [16]	63%	78.8%	39.9%	22.1%	482	13,663	53,248	1251
SiameseRF+rule distillation [19]	57.2%	79.4%	28.2%	23.5%	2000	7265	68,860	2520
Tracktor++ [50]	54.4%	78.2%	19%	36.9%	682	3280	79,149	1480
TPM [51]	51.3%	75.2%	18.7%	40.8%	569	2701	85,504	707
**Ours**	52.2%	77.6%	21.3%	31%	1209	8214	41,514	1311

**Table 5:** Time consumption of collector and filter with different batch sizes.

	Batch Size = 1	Batch Size = 5	Batch Size = 10	Batch Size = 15
Collector (C)	0.147 ms	0.599 ms	1.211 ms	1.694 ms
Filter (F)	0.289 ms	0.744 ms	1.047 ms	1.361 ms

## Data Availability

The raw/processed data required to reproduce these findings cannot be shared at this time as the data also forms part of an ongoing study.
